# Clinical evaluation of premonitory urges in children and adolescents using the Chinese version of Individualized Premonitory Urge for Tics Scale

**DOI:** 10.3389/fpsyt.2023.1224825

**Published:** 2023-11-16

**Authors:** Guanghua Che, Wenjing Ren, Joseph F. McGuire, Ping Li, Zhiruo Zhao, Jing Tian, Jinyuan Zhang, Yue Zhang

**Affiliations:** ^1^Department of Developmental Pediatrics, The Second Hospital of Jilin University, Changchun, China; ^2^Pediatrics Centre, The Second Hospital of Jilin University, Changchun, China; ^3^Center for Obsessive-Compulsive Disorder (OCD), Anxiety, and Related Disorders for Children, Division of Child and Adolescent Psychiatry, Department of Psychiatry and Behavioral Sciences, Johns Hopkins University School of Medicine, Baltimore, MD, United States

**Keywords:** tic disorders, premonitory urges, Premonitory Urge for Tics Scale, Individualized Premonitory Urge for Tics Scale, reliability, validity

## Abstract

**Background:**

Premonitory urges (PUs) have been the focus of recent efforts to assess the severity and develop interventions for tic disorders (TD). We aimed to investigate the PUs in TD and its comorbidities from multiple dimensions, using the Chinese version of the Premonitory Urge for Tics Scale (C-PUTS) and the Chinese version of the Individualized Premonitory Urge for Tics Scale (C-IPUTS), in order to provide perspectives for the diagnosis and management of TD in children and adolescents.

**Methods:**

A total of 123 cases were included in the study. The IPUTS was translated, back-translated, culturally adjusted, and pre-investigated to determine the items of the C-IPUTS. The reliability and validity of the C-IPUTS scale were evaluated by a questionnaire survey on children and adolescents with TD at the Developmental Pediatrics Department of the Second Hospital of Jilin University. Meanwhile, the C-PUTS, which had been evaluated and used in China, Yale Global Tic Severity Scale (YGTSS), Yale-Brown Obsessive-Compulsive Scale (Y-BOCS), Depression Self-Rating Scale (DSRS), Screen for Childhood Anxiety-Related Disorders (SCARED), Achenbach Child Behavior Checklist (CBCL), and Swanson, Nolan and Pelham, Version IV (SNAP-IV), were used to assess the association of PUs with tics and comorbidities of TD.

**Results:**

All dimensions of the C-IPUTS demonstrated good reliability and validity. Our findings suggested that PUs in children and adolescents in China occurred primarily at the head/face and neck/throat. The different dimensions of the C-IPUTS (number, frequency, and intensity) and C-PUTS were positively correlated with the YGTSS total score, while the C-PUTS was positively correlated with the Y-BOCS, SCARED, DSRS, and SNAP-IV scale total scores. The three dimensions of the C-IPUTS demonstrated correlations with anxiety severity and obsessive-compulsive symptoms.

**Conclusion:**

The C-IPUTS can be used to assess PUs reliably and effectively and provide further information for the C-PUTS from various dimensions in a Chinese setting. PUs relate to obsessive-compulsive symptoms, anxiety, attention deficit hyperactivity, and behavioral problems in children and adolescents with TDs. Accordingly, PUs evaluation using the C-IPUTS combined with the PUTS might provide useful information for future therapies for TDs to achieve greater tic reduction.

## 1. Introduction

Premonitory urges (PUs), often described as aversive or unpleasant sensations (1), are sensory phenomena preceding the onset of tics (2) in tic disorders (TD). PUs are often described by patients as “uncomfortable itching,” “sneezing,” (2) and sensations such as “energy release sensation,” “pressure sensation,” and “itching” or “pain” before tics occur. Leckman et al. (3) pointed out that PUs are the core symptoms of TDs, and can be more painful and/or uncomfortable than tics themselves in some cases. While the nature of tics is multi-factorial, tics are often preceded by PUs and tic expression reportedly temporarily alleviates the discomfort from PUs. Cavanna et al. (4) showed that PUs, and tic severity of TDs can be used as predictors of health-related wellbeing in late adolescence and adulthood. Previous meta-analyses in China showed that PUs have a strong correlation with TDs and play a key role in the expression of tic symptoms (5).

Aside from tics themselves, PUs are associated with attention deficit hyperactivity disorder (ADHD), anxiety, depression, and obsessive-compulsive symptoms (OCS) (6). Similar to the adverse effects of comorbid conditions and coexisting psychopathology on patients’ quality of life, PUs may be more troublesome than tics themselves. In addition, the severity of PUs can predict a reduction in the benefit of behavioral therapy (7), and serves as a potential treatment mechanism underlying tic severity reductions (8, 9). The recommended first-line behavioral interventions for TD, including habit reversal training (HRT) and exposure and defense response (ERP), are based on the perception and habituation to PUs (10). Based on these conditions, it is recommended that PUs could be considered as a target in the assessment and management of tics.

Premonitory urges evaluation has been one of the focuses of the assessment and management of TDs in recent years [see McGuire et al. (11) for the recommendation of evidence-based assessment of TD].Despite the intrinsic sensation of PUs, multiple TDs, as well as individual differences exist in PUs. One self-report checklist, the Premonitory Urge for Tic Scale (PUTS) (12), has been widely used to measure PUs in patients with TD in the United States (12), China (13), Italy (14), Spain (15), Japan (16), and South Korea (17). Rajagopal et al. (18) found that the PUTS could distinguish between types of PUs associated with simple or complex tics as well as obsessive phenomena. However, the following issues with the PUTS warrant further investigation on PUs. First, the PUTS takes PUs as a whole construct, measured over an uncertain period, and does not enable subjects to identify differences in between specific urges toward different tics. Second, the analysis of the PUTS is limited to an individual dimension of all urges, which does not include evaluation of the frequency and intensity of the urges (19).

An alternative and complementary approach to assessing PUs are necessary. Different individuals and tics have been reported to have different degrees of PUs (20), and individualized urge evaluation might provide crucial supplementary details for the assessment of PUs and provide the opportunity to assess urges from multiple dimensions (21). To this end, McGuire et al. (22) developed the Individualized Premonitory Urge for Tics Scale (I-PUTS) and confirmed that the I-PUTS is a reliable and effective phenomenological measurement of urge that can capture multifarious dimensions of PUs, which provides complete information for PUs and assesses body regions associated with PUs, further advancing understanding of the phenomenology. Nevertheless, the Chinese version of the I-PUTS has not yet been published, and the psychological properties of a Chinese version of the I-PUTS need to be further established.

The comorbid conditions and coexisting psychopathology (e.g., anxiety disorders, OCS, and ADHD) might influence youth with TD (23). Evidence showed correlation of varying strengths between the PUs and OCS (16), overall anxiety symptoms (24), and depressive symptoms (22). As for the associations between ADHD severity and PUs, inconsistent conclusions exist (25). On the other hand, I-PUTS could distinctly capture PUs phenomena, and its ratings were not significantly influenced by co-occurring psychopathology (22). Comparatively, the PUTS total score exhibited a large negative relationship with distress tolerance and a moderate-to-large positive correlation with anxiety severity (22). Therefore, this study will help illustrate the relationship of comorbid conditions and PUs assessment.

This study aimed to assess the psychometric properties of the Chinese version of the I-PUTS (C-IPUTS) and investigate the multiple sites of PUs by combining the self-report PUTS with the clinician-administered C-IPUTS in a Chinese setting. We also explored the network correlation between C-IPUTS dimensions, total C-PUTS score, tics, and other clinical properties (including obsessive-compulsive behavior, ADHD, anxiety, and depression), which reflects a multidimensional and more comprehensive assessment of PUs.

## 2. Materials and methods

### 2.1. Participants

This study initially enrolled 160 children and adolescents with TDs (based on the Diagnostic and Statistical Manual of Mental Disorders, Fifth Edition, DSM-5) aged between 8 and 14 years old. Children and adolescents with infections, intellectual disability, language impairment, autism spectrum disorder, and major psychiatric disorders (such as schizophrenia and bipolar disorder) were excluded. After detailed notification of the protocol, at least one caregiver and their child signed an informed consent. In total, 123 youth ultimately participated in this study. All participants were enrolled from the Department of Developmental Pediatrics of the Second Hospital of Jilin University from September 2021 to August 2022. None of the participant were receiving any tic-influencing medication when enrolled in the study.

### 2.2. Scales for assessments

#### 2.2.1. Assessment of premonitory urge using C-PUTS and C-IPUTS

The C-PUTS is a self-report question sheet that measures PUs in patients with TDs (12). C-PUTS has demonstrated reliability and validity in assessing PUs in Chinese patients between 6 and 16 years old with TDs (13). The total score varies from 9 to 36 points, and higher scores mean greater severity of PUs.

The I-PUTS (22) is a clinician-administered measurement that assesses the existence, frequency, intensity, and body parts with recognized PUs in the past week. Before performing the I-PUTS translation, we obtained an agreement from Dr. McGuire. We performed an initial and forward-backward translation of the I-PUTS to Chinese in accordance with the guidelines proposed by Beaton et al. (26). The physician asked the participant whether there was a PUs before the tic and rated the corresponding score on a 4-point scale for each recognized tic. Items were scored 0 when tics or PUs are not recognized. Then, the physician requested information about the body parts related to each PUs. Eventually, the data were pooled to obtain the total number, frequency, and intensity of the urges and the site of PUs occurrence.

#### 2.2.2. Yale Global Tic Severity Scale

The Yale Global Tic Severity Scale (YGTSS) (27) is a clinician-completed semi-structured interview rating scale that quantifies the severity and specific nature, including number, frequency, intensity, complexity, and interference, of motor and vocal tics. The YGTSS also provides an impairment score that focuses primarily on the impact of TDs on self-esteem, family life, social acknowledgment, or school in the last week. The maximum total YGTSS score is 100, including a maximum tic severity score of 50 (each half for motor and vocal tics) and the other 50 for the tics impairment. The YGTSS has been demonstrated to have satisfactory reliability and validity and is extensively used internationally and in China.

#### 2.2.3. The Yale-Brown Obsessive Compulsive Scale

The Yale-Brown Obsessive Compulsive Scale (Y-BOCS) (28) is a clinician-rated, semi-structured instrument consisting of 10 items to assess the severity of obsessive and compulsive symptoms over the past week. The validity and reliability of this scale have been well documented in many studies and is suitable for children and adults aged 8 years and older (29).

#### 2.2.4. Achenbach Child Behavior Checklist

The Achenbach Child Behavior Checklist (CBCL) (30) is a parent-report eight-subscale questionnaire with excellent psychometrics. It is mainly used to screen children and adolescents for social ability and behavioral problems, and the social ability part is only used for those aged 6–18 years; behavioral problems are divided into four age/gender groups, that is, boys and girls aged 4–11 and 12–18 years. The behavior problem part is composed of 113 question items, of which the 56th question includes 8 question items (56a–56h), with a total of 120 questions. Each question item is scored according to a three-level score of 0–2: 0 means no problem, 1 means a mild or occasional problem, and 2 means an obvious or frequent problem. In the present study, the Total Problems, Internalizing Problems (Anxious/Depressed, Withdrawn, and Somatic Complaints), and Externalizing Problems (Rule Breaking Behavior and Aggressive Behavior) scales scores from the CBCL were used to estimate behavioral and emotional problems.

#### 2.2.5. Depression Self-Rating Scale for Children

The Depression Self-Rating Scale for Children (DSRS-C) (31) can be used as a cost-effective screener for depression in children and adolescents aged 8–14, with sound psychometric properties in clinical samples. The 18-item self-rating scale is scored on three levels: none (0), sometimes (1), and often (2). Depression is considered when the total score is 13 or more. Higher scores represent stronger depressive trend.

#### 2.2.6. Screen for Childhood Anxiety-Related Disorders

The 41-item Screen for Childhood Anxiety-Related Disorders (SCARED) (32) is a measure widely used for the assessment of recent anxiety symptoms in those aged 6–18 based on parent and child reports, including SCARED parent and child versions. Participants respond on a 3-point scale of 0, 1, or 2. What is more, five-subscale symptoms generalized anxiety, separation anxiety, social anxiety, panic or somatic symptoms, and school avoidance are included. A total score of 23 or above indicates clinical anxiety.

#### 2.2.7. Swanson, Nolan and Pelham, Version IV

The Swanson, Nolan and Pelham, Version IV (SNAP-IV) parent rating scale consists of 26 items and includes three dimensions: impulsivity/hyperactivity, inattention, and oppositional defiant symptoms. The Chinese SNAP-IV was proved as a reliable and valid tool for assessing ADHD-related symptoms of children and adolescents aged 6–15 years in clinical settings. It has good reliability and validity and psychometric properties in China (33). Each dimension score is divided by the item number which includes 9 for inattention and hyperactivity-impulsivity and 8 for oppositional defiant symptoms. The inattention subset and hyperactivity/impulsivity subset of the parent-rated cut-off are 1.78 and 1.74, respectively.

### 2.3. Procedures

The study protocol was approved by the ethics committee of the Second Hospital of Jilin University. Two developmental behavioral pediatricians independently conducted the assessments. Informed consent was obtained from all participants and their caregivers prior to participation. Sociodemographic data were collected via questionnaires. Participants and parents completed clinician-administered measures to assess tic severity (YGTSS), PUs phenomenology (C-IPUTS), and obsessive and compulsive symptoms (Y-BOCS). Parents also completed the CBCL, SNAP-IV, and SCARED-P rating scales, while youth completed the PUTS, SCARED-C, and DSRS-C self-report scales. To establish inter-rater reliability, a second rater conducted retest of the C-IPUTS and C-PUTS 1 month after the first assessment.

### 2.4. Network analysis

Network analysis was used to analyze the relationships among these factors and the structures of scales arising from the recurring associations. A network graph was constructed based on the total sample, incorporating the number, frequency, and intensity dimensions from the C-IPUTS; the impairment and severity of motor and vocal tics from the YGTSS, and C-PUTS, CY-BOCS, SCARED, DSRS, CBCL, and SNAP-IV scales. Solid lines in the graph represent positive correlations, while dashed lines illustrates negative correlations. The thickness of each line correlates with the *P*-value (thicker lines indicate smaller *P*-values and greater *r* values). The line color intensity correlates with the absolute *r* value. R software (version 3.2.2^1^) and Cytoscape were used to estimate networks and visualize correlations.

### 2.5. Statistical methods

Statistical analyses were completed using SPSS software (v26, IBM Corp., Armonk, NY, USA). Descriptive statistics characterized the PUs and clinical characteristics based on scales from the I-PUTS and other scales. First, we performed a normality test. Since the I-PUTS dimensions and PUTS total scores were not normally distributed, non-parametric statistics were used. Kruskal–Wallis tests compared differences in C-IPUTS urge number, frequency, and intensity across body regions. Second, we used the frequency and constituent ratio to describe sociodemographic data and the Kruskal–Wallis *H* test to compare differences in frequency and intensity in each body region. Third, we used reliability analysis (reliability, test-retest reliability) to test the internal consistency and temporal stability of the C-IPUTS. Interclass correlation coefficients (ICCs) were used to evaluate inter-rater reliability for each I-PUTS dimension. Fourth, we used exploratory factor analysis (EFA) to analyze the validity of the C-IPUTS. Fifth, we used Spearman correlation analysis to analyze the correlation between the C-IPUTS (number of urges, frequency, and intensity) and C-PUTS; convergent validity was verified by analyzing each dimension of the C-PUTS, C-IPUTS (number of urges, frequency, and intensity), and YGTSS. Sixth, we used correlation analysis to analyze the relationship between each dimension of the C-IPUTS, C-PUTS, and other comorbidities. Finally, the children and adolescents were divided into two groups: who exhibited concordance on the C-IPUTS and C-PUTS (i.e., reported the consistent presence or absence of PUs on both scales), and those who exhibited discordance (i.e., reported PUs on one scale but not the other). An independent samples *t*-test was used to assess whether there were statistical differences between youth with concordant versus discordant scores, and to evaluate whether other symptoms were present for those with discordant C-IPUTS and C-PUTS scores.

## 3. Results

### 3.1. Clinical characteristics of participants

The age of participants ranged from 8 to 14 years, with a median age of 10 (9, 11), including 99 boys with a median age of 10 (9, 12) and 24 girls with a median age of 9 (8, 10.5). To perform the comparison analysis with the published literature (12), the participants were divided into two groups: 8–10 years (younger age group) (*n* = 73) and 11–14 years (older age group) (*n* = 50). There were 55 boys (75.34%) and 18 girls (24.66%) in the younger age group and 45 boys (90%) and 5 girls (10%) in the older age group. The most common site of the first tic was the eyes, accounting for 51.22%. The most common comorbidity was ADHD (*n* = 48, 39.02%), followed by OCS (*n* = 25, 20.33%), anxiety disorders (*n* = 23, 18.70%), depression (*n* = 19, 15.45%), and behavioral problems (*n* = 17, 13.82%). Finally, 11.38% of participants revealed a family history of tics.

### 3.2. Premonitory urges characteristics

Among the participants, 86.99% (n = 107) cases reported PUs for endorsed tics on the I-PUTS, while 84.55% (n = 104) youth reported PUs on C-PUTS. On average, youth confirmed 7 tics over the past week (Mean = 7.35, SD = 4.25), and experienced PUs 50–75% of the time they had the tic (Mean = 3.70, SD = 2.67), rating the intensity as moderate (Mean = 3.48, SD = 2.36). PUs were predominantly localized in the head/face region (53.3%), neck/throat region (17.3%), arms (7.0%), legs (6.3%), torso (11.0%) and whole body/other regions (5.1%). Kruskal–Wallis *H* tests revealed significant differences in PUs number (χ^2^ = 20.67, *P* = 0.001), frequency (χ^2^ = 21.34, *P* = 0.001), and intensity (χ^2^ = 17.55, *P* = 0.004) across body regions, with higher intensity ratings for PUs from the neck/throat versus other regions (Table 1).

**TABLE 1:** Mean, standard deviation, median, and interquartile range for C-IPUTS dimensions by urge body region, and pair-wise comparisons for average frequency and intensity ratings.

	Mean (SD)	Range	1	2	3	4	5
**Average C-IPUTS frequency**							
1. Head and face region	3.42 (1.95)	1–4					
2. Neck and throat region	3.53 (1.56)	1–4	0.380				
3. Arms	3.16 (1.73)	1–4	0.46	0.76			
4. Legs	2.25 (1.52)	1–4	0.97	0.75	0.39		
5. Torso	2.92 (1.15)	1–4	0.69	1.42	0.33	0.77	
6. Whole body and other region	2.23 (1.17)	1–4	4.56***	4.39***	1.43	0.434	0.888
**Average C-IPUTS intensity ratings**							
1. Head and face region	3.55 (2.45)	1–4					
2. Neck and throat region	3.61 (2.68)	1–4	1.25				
3. Arms	3.14 (1.57)	1–4	3.82**	4.17***			
4. Legs	2.75 (1.25)	1–4	5.49***	5.53***	0.25		
5. Torso	3.23 (1.58)	1–4	3.29*	3.71**	0.26	0.53	
6. Whole body and other region	3.05 (1.71)	1–4	4.45***	4.04***	1.35	1.58	1.33

*P < 0.05, **P < 0.01, and ***P < 0.001.

### 3.3. Reliability and validity of the C-IPUTS and other scales

The Cronbach’s α coefficient of the total C-IPUTS scale was 0.899, including 0.899 in the younger age group and 0.902 in the older age group, all of which were greater than 0.70, indicating that the C-IPUTS had good internal consistency. A total of 115 cases (93.50%) completed the 1-month follow-up with the C-IPUTS demonstrating good test-retest reliability (ICC = 0.910). This is consistent with previous findings from McGuire et al. (22). The mean Kaiser–Meyer–Olkin (KMO) score for the 123 participants was 0.784, indicating that the factor analysis model was suitable for this data and factor analysis could be performed. The approximation of Bartlett’s test of sphericity was 458.135, with 3 degrees of freedom (*P* < 0.001), indicating the applicability of the factor analysis model. The C-IPUTS explained 94.082% of the total variance and showed good construct validity. The Cronbach’s α coefficients for the C-PUTS, YGTSS motor and phonic tic subscales, YGTSS impairment and total scores, Y-BOCS, SCARED, DSRS, SNAP-IV, and CBCL internalizing, externalizing, and total scales were almost all above 0.7, indicating good internal consistency (Table 2). Inter-rater reliability was also good based on interclass correlation coefficients (ICCs) for the I-PUTS Urge Number (0.863), Frequency (0.837), and Intensity (0.694) subscales, as well as the YGTSS (0.981) and Y-BOCS (0.918) total scores.

**TABLE 2:** Summary of clinical assessments (*n* = 123).

Scale	Mean	SD	*M*	(q25, q75)	Range	Items	Cronbach’s α
C-PUTS first	15.44	4.285	15	(13, 18)	9–26	9	0.757
C-PUTS second	15.25	4.128	15	(13, 18)	9–24	9	0.755
C-IPUTS total score first	2.92	2.651	2	(1, 4)	0–14	46*3	0.899
C-IPUTS number first	1.49	1.418	1	(1, 2)	0–6	46	0.740
C-IPUTS frequency first	3.80	3.169	3	(2, 5)	0–14	46	0.803
C-IPUTS intensity first	3.46	2.584	3	(2, 4)	0–11	46	0.944
C-IPUTS total score second	2.28	2.110	2	(1, 3)	0–12	46*3	0.914
C-IPUTS number second	1.49	1.148	1	(1, 2)	0–5	46	0.825
C-IPUTS frequency second	3.64	3.133	3	(2, 5)	0–11	46	0.931
C-IPUTS intensity second	3.22	2.207	3	(2, 4)	0–11	46	0.722
YGTSS motor tics	7.43	3.865	9	(5, 10)	0–17	5	0.786
YGTSS phonic tics	2.92	2.993	3	(0, 5)	0–11	5	0.792
YGTSS impairment	16.18	7.302	10	(10, 20)	10–40	1	0.923
YGTSS total	26.57	9.800	25	(19, 33)	13–62	11	0.707
Y-BOCS	2.54	3.694	1	(0, 3)	0–14	10	0.766
SCARED	14.95	8.955	14	(8, 20)	0–46	41	0.776
DSRS	8.36	4.551	8	(5, 11)	0–20	18	0.706
SNAP-IV-1	0.80	0.65	0.7	(0.3, 1.1)	0–2.8	9	0.913
SNAP-IV-2	0.62	0.552	0.6	(0.1, 1)	0–2.1	9	0.899
SNAP-IV-3	0.64	0.483	0.6	(0.3, 0.9)	0–2.4	8	0.882
CBCL internalizing scale	6.56	6.130	5	(2, 9)	0–26	36	0.695
CBCL externalizing scale	8.07	7.126	6	(3, 11)	0–34	30	0.716
CBCL total score	26.05	18.556	21	(11, 37)	0–83	113	0.771

### 3.4. Correlations between the I-PUTS, PUTS, and clinical data

**FIGURE 1:**
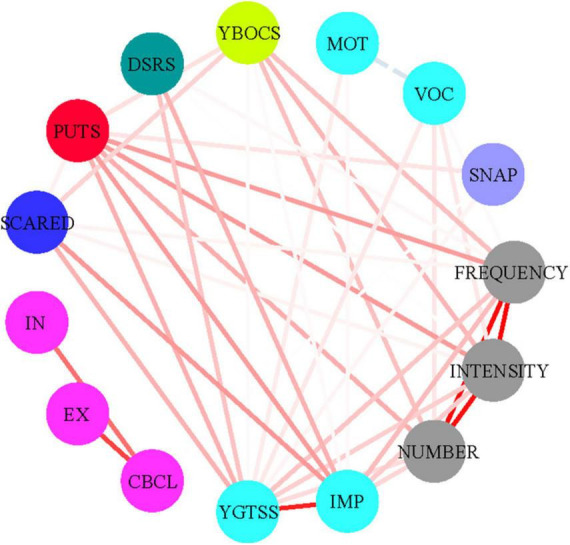
Correlations between the PUTS and I-PUTS scores and clinical constructs. PUTS, Premonitory Urge for Tics Scale; NUMBER, number item of I-PUTS; FREQUENCY, frequency item of I-PUTS; INTENSITY, intensity item of I-PUTS; VOC, vocal subscale of YGTSS; MOT, motor subscale of YGTSS; IMP, impairment item of YGTSS; YGTSS, Yale Global Tic Severity Scale; Y-BOCS, Yale-Brown Obsessive Compulsive Scale; SCARED, Screen for Childhood Anxiety-Related Disorders; DSRS, Depression Self-Rating Scale; SNAP-IV, Swanson, Nolan and Pelham, Version IV; CBCL, Achenbach Child Behavior Checklist; IN, CBCL internalizing scale; EX, CBCL externalizing scale.

Table 3 and Figure 1 demonstrate correlations between the number, frequency, and intensity of C-IPUTS PUs and the total C-PUTS score (*P* < 0.05). The C-IPUTS and C-PUTS scales showed a strong positive relationship for premonitory urge symptoms. In particular, robust correlations existed among the three C-IPUTS dimensions assessing PUs. In TD, the number, frequency, and intensity of urges positively correlated with the YGTSS vocal tic scale score and C-IPUTS impairment (*P* < 0.05). The C-PUTS also positively correlated with the YGTSS total score, vocal tic scale, and impairment (*P* < 0.05). For TD’s comorbidities, the C-IPUTS better correlated with the Y-BOCS than the SCARED, but showed no correlations with the SNAP-IV, DSRS, or CBCL. The C-PUTS correlated with the Y-BOCS and SCARED, DSRS, and SNAP-IV scales, but not the CBCL.

**TABLE 3:** Spearman correlation between scales (*n* = 123).

	C-IPUTS total number	C-IPUTS total frequency	C-IPUTS total intensity	C-PUTS total score
C-PUTS total score	0.433**	0.486**	0.489**	–
YGTSS total motor tic score	0.017	0.116	0.084	0.055
YGTSS total phonic tic score	0.271**	0.215*	0.267**	0.151
YGTSS impairment	0.317**	0.394**	0.349**	0.483**
YGTSS total tic score	0.301**	0.370**	0.339**	0.427**
**Comorbid symptom severity**				
Y-BOCS total score	0.382**	0.387**	0.411**	0.288**
SCARED total score	0.189*	0.230*	0.231*	0.239*
DSRS total score	0.173	0.221*	0.203*	0.165
SNAP-IV total score	0.134	0.161	0.155	0.274**
CBCL total score	-0.033	-0.033	-0.025	0.011
CBCL internalizing scale	-0.138	-0.098	-0.141	-0.204*
CBCL externalizing scale	-0.112	-0.100	-0.128	-0.188

*P < 0.05, **P < 0.01.

C-IPUTS, the Chinese version of the Individualized Premonitory Urge for Tics Scale; C-PUTS, the Chinese version of the Premonitory Urge for Tics Scale; YGTSS, Yale Global Tic Severity Scale; Y-BOCS, Yale-Brown Obsessive-Compulsive Scale; SCARED, Screen for Childhood Anxiety-Related Disorders; DSRS, Depression Self-Rating Scale; SNAP-IV, Swanson, Nolan and Pelham, Version IV; CBCL, Achenbach Child Behavior Checklist.

In our sample, the male to female ratio was 4.3:1. The mean C-PUTS score was 16 (13, 18) for males and 14 (13, 17) for females. However, rank sum tests indicated these differences were not statistically significant (*P* > 0.05). Moreover, no significant differences existed between boys and girls for the C-IPUTS number, frequency, or intensity of PUs (*P* > 0.05). No significant correlations were found between age and C-PUTS or C-IPUTS scores among the total sample.

### 3.5. Correlation between scales in different age groups

Retrospective reports indicate that PUs typically emerge around the ages 6–7 years (34) but may not be detected until ages 8–10 years (3) as children may lack the ability to recognize and describe urge awareness before age 8 (35). Furthermore, several studies have demonstrated an effect of age using the PUTS and clinician assessments using the I-PUTS (6). To further elucidate this relationship, participants in the current study were divided into two age groups – 8–10 years (younger) and 11–14 years (older).

We assessed the correlations between the C-IPUTS, C-PUTS total scores, TD, and comorbidities of TD in different age groups using Spearman correlation analysis. The results showed that there were mild to moderate correlations between the C-IPUTS score and the C-PUTS, YGTSS, Y-BOCS, SCARED, and SNAP-IV scores in the younger age group (*P* < 0.05). There were no significant correlations between the C-IPUTS/C-PUTS scores and the DSRS or CBCL score (*P* > 0.05). In the older age group, the C-IPUTS score showed mild correlations with the YGTSS (*r* = 0.338–0.412, *P* = 0.003–0.016 < 0.05) and C-PUTS (*r* = 0.283–0.331, *P* = 0.028–0.046 < 0.05) scores, but was not significantly associated with any of the other scales (*r* = 0.028–0.269, *P* = 0.059–0.857 > 0.05). The C-PUTS score in the younger age group had mild to moderate correlations with the YGTSS, Y-BOCS, SCARED, and SNAP-IV scores (*P* < 0.05) but had no significant correlations with the DSRS or CBCL score (*P* > 0.05). In the older age group, C-PUTS was not significantly associated with other scales (*r* = 0.012–0.277, *P* = 0.051–0.947 > 0.05), except for YGTSS (*r* = 0.427, *P* < 0.01). The correlation analyses in the younger age group and older age group are detailed in Tables 4, 5, respectively.

**TABLE 4:** Correlation between scales in the younger age groups (*n* = 73).

	C-IPUTS total number	C-IPUTS total frequency	C-IPUTS total intensity	C-PUTS total score
PUTS total score	0.526**	0.601**	0.600**	–
YGTSS total motor tic score	-0.057	0.073	0.063	0.030
YGTSS total phonic tic score	0.373**	0.309**	0.327**	0.258*
YGTSS impairment	0.298*	0.381**	0.384**	0.506**
YGTSS total tic score	0.246*	0.339**	0.333**	0.427**
**Comorbid symptom severity**				
Y-BOCS total score	0.454**	0.463**	0.468**	0.360**
SCARED total score	0.254*	0.275*	0.296*	0.266*
DSRS total score	0.105	0.164	0.171	0.112
SNAP-IV total score	0.221	0.295*	0.265*	0.355**
CBCL total score	-0.030	0.001	-0.052	-0.030
CBCL internalizing scale	0.179	0.176	0.144	0.123
CBCL externalizing scale	0.230*	0.254*	0.231*	0.153

*P < 0.05, **P < 0.01.

C-IPUTS, the Chinese version of the Individualized Premonitory Urge for Tics Scale; C-PUTS, the Chinese version of the Premonitory Urge for Tics Scale; YGTSS, Yale Global Tic Severity Scale; Y-BOCS, Yale-Brown Obsessive-Compulsive Scale; SCARED, Screen for Childhood Anxiety-Related Disorders; DSRS, Depression Self-Rating Scale; SNAP-IV, Swanson, Nolan and Pelham, Version IV; CBCL, Achenbach Child Behavior Checklist.

**TABLE 5:** Correlation between scales in the older age groups (*n* = 50).

	C-IPUTS total number	C-IPUTS total frequency	C-IPUTS total intensity	C-PUTS total score
PUTS total score	0.283*	0.304*	0.600**	–
YGTSS total motor tic score	0.129	0.180	0.130	0.094
YGTSS total phonic tic score	0.109	0.097	0.179	-0.010
YGTSS impairment	0.394**	0.414**	0.308**	0.455**
YGTSS total tic score	0.390*	0.412**	0.338**	0.497**
**Comorbid symptom severity**				
Y-BOCS total score	0.258	0.269	0.307*	0.170
SCARED total score	0.119	0.176	0.141	0.194
DSRS total score	0.291*	0.337*	0.260	0.277
SNAP-IV total score	-0.048	-0.028	-0.029	0.158
CBCL total score	-0.043	-0.057	-0.052	0.127
CBCL internalizing scale	-0.067	0.020	-0.146	-0.149
CBCL externalizing scale	-0.075	-0.014	-0.114	0.005

*P < 0.05, **P < 0.01.

C-IPUTS, the Chinese version of the Individualized Premonitory Urge for Tics Scale; C-PUTS, the Chinese version of the Premonitory Urge for Tics Scale; YGTSS, Yale Global Tic Severity Scale; Y-BOCS, Yale-Brown Obsessive-Compulsive Scale; SCARED, Screen for Childhood Anxiety-Related Disorders; DSRS, Depression Self-Rating Scale; SNAP-IV, Swanson, Nolan and Pelham, Version IV; CBCL, Achenbach Child Behavior Checklist.

### 3.6. Agreement and inconsistency between C-IPUTS and C-PUTS

Several children and adolescents with TDs and PUs had inconsistent results between the C-IPUTS and C-PUTS. For example, 16 children and adolescents reported PUs on the self-reported C-PUTS which were not confirmed on the C-IPUTS. Similarly, 19 children and adolescents did not report PUs on the C-PUTS, but PUs were identified by clinicians using the C-IPUTS. Participants were divided into concordant (*n* = 100) and discordant (*n* = 23) groups based on agreement between the C-IPUTS and C-PUTS. Mann–Whitney *U* tests showed significant differences in YGTSS total score, phonic tic subscale, and Y-BOCS between the two groups. However, no significant differences were found for SCARED, DSRS, SNAP-IV, and CBCL scores (Table 6).

**TABLE 6:** Characteristics of concordance (*n* = 100) and discordance (*n* = 23) between the C-IPUTS and C-PUTS [*M* (P25, P75)].

Demographic and clinical characteristics	Good agreement (*n* = 100)	Poor agreement (*n* = 23)	*Z*	*P*
Age	10 (9, 11)	9 (8, 11)	-0.904	0.336
**Tic severity and impairment**				
YGTSS total score	28 (19.25, 33.75)	21 (18, 24)	-3.142	0.002
YGTSS total motor tic score	8.5 (4, 10)	9 (7, 10)	-1.364	0.173
YGTSS total phonic tic score	3 (0, 5)	0 (0, 4)	-1.994	0.046
YGTSS impairment	20 (10, 20)	10 (10, 10)	-3.580	<0.001
**Comorbid symptom severity**				
Y-BOCS total score	1 (0, 4)	0 (0, 2)	-2.103	0.035
SCARED total score	14 (11, 21)	10 (5, 14)	-1.359	0.174
DSRS total score	8 (5, 11.75)	6 (4, 9)	-0.990	0.322
SNAP-IV total score	1 (1, 2)	1 (1, 2)	-1.590	0.112
CBCL total score	21.5 (12, 37)	18 (3, 36)	-1.215	0.224
CBCL internalizing scale	5 (2, 10)	4 (2, 8)	-0.761	0.446
CBCL externalizing scale	6 (3, 11)	7 (1, 11)	-0.682	0.495

C-IPUTS, the Chinese version of the Individualized Premonitory Urge for Tics Scale; C-PUTS, the Chinese version of the Premonitory Urge for Tics Scale; YGTSS, Yale Global Tic Severity Scale; Y-BOCS, Yale-Brown Obsessive Compulsive Scale; SCARED, Screen for Childhood Anxiety-Related Disorders; DSRS, Depression Self-Rating Scale; SNAP-IV, Swanson, Nolan and Pelham, Version IV; CBCL, Achenbach Child Behavior Checklist.

## 4. Discussion

This study is the first to examine the feasibility of the I-PUTS for assessing children and adolescents’ PUs in a Chinese cultural context. First, we verified the reliability and validity of the C-IPUTS for Chinese children and adolescents, evaluated the PUs, and compared it with the C-PUTS. Second, this study demonstrated the correlation between PUs using the C-PUTS and C-IPUTS with the tics and other comorbidities.

Reliability of the scale refers to the consistency and stability of the measured results. Cronbach’s α coefficient is commonly used to represent the internal consistency of the scale, and retest reliability is used to represent the external consistency of the scale. The results indicate that the C-IPUTS is a highly readable, simple, and understandable PUs assessment tool with good reliability and validity. The total sample (Cronbach’s α = 0.899) and two age groups (Cronbach’s α = 0.902 in the older age group and 0.889 in the younger age group) had good reliability, representing a stable internal consistency across the age span. The retest reliability of the C-IPUTS was 0.910 1 month after the first assessment. It shows that C-IPUTS has good internal consistency and external consistency.

Factor analysis is the most commonly used method to test the scale structure. EFA is used to determine the scale’s structural validity. EFA revealed that the C-IPUTS had good construct validity with loading >0.40 and cumulative variance explained >40%. When one factor was extracted, the cumulative total variance interpretation rate was 94.082%, indicating that the C-IPUTS had good validity.

This study proposed to further the research by Woods et al. (12) and McGuire et al. (22) by appraising the psychometric properties of the C-PUTS and C-IPUTS in the Chinese context. The C-PUTS score reflects the self-rated PUs, while the C-IPUTS score demonstrates the frequency and intensity of PUs assessed by clinicians. The C-PUTS has been demonstrated to have steadfast reliability, validity, and psychometric properties in China, so we directly compared them in this study (36). This study demonstrated a positive correlation between the C-PUTS and various dimensions of the C-IPUTS, indicating the consistency of the evaluation results of the two scales. The C-PUTS is a trustworthy and valid tool for quantifying PUs, and the instrument is internally consistent and temporally stable. It can be used to assess the severity of PUs in children and adolescents with TD. The C-PUTS is concise and can be completed independently by children. However, they have some limitations. First, the C-PUTS has been reported to show reliable validity and reliability in children and adolescents (5). Nevertheless, many TD patients younger than 8 years in China find it challenging to read and understand the self-checklists, which may limit the applicability of the instrument. Second, the regions of the PUs on the body are not evaluated by the C-PUTS, thus confining the application of the C-PUTS in communicating satisfactorily with the patient. Third, the C-PUTS restricts analysis to a single dimension. The C-IPUTS has several considerations that further refine the C-PUTS. First, the C-IPUTS measures the location of PUs, which complements the existing scale. Second, the C-IPUTS measures PUs as a whole frame across a specific period, and the respondent are not capable to distinguish between individual urges for different tics. As different tics and different individuals have changing degrees of PUs, an individualized urge evaluation may offer important supplemental information and provide the opportunity to assess urges from various aspects. Both complement each other and provide good tools for assessing PUs.

In this study, the main sites of PUs were the head/face and throat/neck. Essing et al. (37) revealed that PUs were predominantly localized in the forehead, cheeks, mouth, and throat. Leckman et al. (3) found that PUs can manifest in any part of the human body, and the symptoms may manifest systemically or locally. Nevertheless, the symptoms often appear in the face, neck, shoulders, arms, and midline of the abdomen (38). Leckman et al. (3) reported PUs awareness arises three years after tic onset. This may indicate the subtle and elusive sensory information can enter conscious awareness owing to maturational shift in the cognitive processing of somatosensory experience. Or this is related with a function of the location and type of tics involved, which is consistent with report with regard to the mean age of tic onset involving those anatomical regions closely linked with PUs to a great extent (3). McGuire et al. (22) showed that the neck/throat region may have specific sensory connections to vocal tics that contribute to youth’s awareness of greater urge intensity. In terms of gender, we found that males and females had no significant difference in their self-reported or parent-reported PUs on the C-PUTS and C-IPUTS, which is similar to the findings of Edwards et al. (39). It has been found that the incidence of TD is high in men, and the age of onset is earlier than that in women, but the complexity of tics in women increases with age (40), and this phenomenon has not been found in the present study.

For the entire sample of 123 children and adolescents, we found that the number, frequency, and intensity of the PUs was positively correlated with tic severity ratings, the vocal tics subscale, and impairment, but was not significantly correlated with motor tics, indicating that youngsters with more severe TD also had more evident PUs. Meanwhile, the number, frequency and intensity of PUs in C-IPUTS were correlated with OCS, anxiety severity, and depression. Compared with C-IPUTS, C-PUTS score was significantly correlated with total YGTSS score and impairment, while C-PUTS score was correlated with OCS, anxiety and ADHD severity. The results indicate that the combined use of C-IPUTS and C-PUTS facilitates the evaluation of PUs across multiple domains including the TD comorbidities. A recent meta-analysis (5) suggests that PUs exhibit a robust association with tics, appear integral to tic expression, and may serve as a marker of tic severity. For instance, one study (2) found that 71% of patients with TD reported that in the absence of PUs, their tics would disappear. As such, these urges could indicate not just the presence of tics but also their clinical severity.

In this study, the C-PUTS score demonstrated more correlations with comorbidities (anxiety severity, ADHD, and OCS) in the younger age group compared with the older age group. Similarly, the C-IPUTS score correlated with more comorbidities of TD (anxiety and ADHD) in the younger versus older group. In the older age group, OCS had a mild correlation with the C-IPUTS. This aligns with findings by Woods et al. (12) but differs somewhat from Raines et al. (41) and McGuire et al. (22). McGuire found no significant C-IPUTS and age correlation, yet a C-PUTS and age correlation existed (12). This may be because of difficulty distinguishing PUs from OCS-related sensory phenomena and internal sensory perception, potentially due to clinicians not asking the right questions or children lacking cognition to differentiate these phenomena. Though this study found no significant relationship between PUs and age, further research is still needed to explore potential associations between the C-IPUTS, C-PUTS, and age.

The findings of this study revealed positive relationships between PUs and symptoms of ADHD, OCS, and anxiety, consistent with previous research (38). Youth with poorer distress tolerance appear particularly prone to more frequent and intense PUs, experiencing them as aversive (22). Indeed, the prevalence of ADHD, anxiety, and OCS over the lifetime is higher among children with TD compared to neurotypical children (42). The most prevalent comorbidity among patients with TD is ADHD. There appears to be significant overlap between PUs and inner restlessness in those with ADHD (43), with severe PUs potentially linked to greater distractibility (44) in this population. A robust association also exists between PUs and OCS. Patients with both TD and OCS engage in repetitive, guilt-driven behaviors until achieving a sense of completeness, reportedly experiencing PUs more intensely during this process compared to neurotypical individuals (8). However, the interrelationship between PUs and compulsive behaviors remains to be clarified. Structural abnormalities in the sensorimotor cortex seem to exist in both OCS (36) and TD (35); nevertheless, it is indefinite whether structural alterations in sensorimotor areas are a cause or a consequence of the urge. In future studies, fMRI and modeling hemodynamic functions (regressors) could be used to find out neural association of urges preceding mental compulsions in TD patients with OCS. In addition, it was reported that about 5% patients with OCS experienced at least one sensory phenomenon (45). Additionally, patients with sensory phenomena were more prone to have comorbid TD than those without sensory phenomena (46).

The three dimensions of the C-IPUTS exhibited correlations with anxiety severity and OCS. Indeed, anxiety and OCS are common co-occurring conditions of TD (47), which are also accompanied by somatosensory sensitivities. This indicates that the I-PUTS substantially captures urge phenomena, and its ratings are not notably affected by co-occurring psychopathology. The co-occurrence of these psychiatric disorders may enhance sensitivity to PUs. Future research should further explore the nature of the PUs experience of TD patients with or without comorbidities and develop tools/methods to assess these experiences in detail.

All 81.30% of participants had concordant results on the clinician-administrated C-IPUTS and self-reported C-PUTS. It is important to note that the assessment timeline for the C-IPUTS is PUs experienced in the past week, while the C-PUTS does not have a well-defined assessment time, and the established association may be greater if the two scales have similar assessment intervals. This may also be responsible for the inconsistency in scores between the two scales. Another reason for the difference between C-IPUTS and C-PUTS scores may be that the children and adolescents in this study had TD and other co-occurring psychiatric symptoms, such as OCS, ADHD, depression, and anxiety, and children and adolescents are liable to have unintentionally confused other uncomfortable somatosensory or somatic anxiety sensations with PUs in their PUTS self-report ratings. Alternatively, children and adolescents may also have unintentionally biased results due to anxiety when completing the C-IPUTS under the guidance of clinicians. The presence of the above problems in children and adolescents may lead to different degrees of deviation in the understanding of the C-PUTS and C-IPUTS, highlighting the importance of using both scales to capture complementary information.

Premonitory urges are a major focus of current research, with many experts positing that they may serve as a marker of TD severity. An Asian study utilized PUTS scales to assess premonitory urge symptoms in adolescents and adults with Tourette’s disorder. After 4 years of treatment, patients’ global functioning was negatively correlated with tics and PUs compared to before treatment. Improvement was associated with reduced severity of OCS and PUs (16). As such, the presence and intensity of PUs across dimensions are critical for effectively treating Tourette’s. When used together, the C-PUTS and C-IPUTS can assess both the occurrence and multidimensional severity of PUs, especially for those with comorbid anxiety and OCS. This represents an invaluable tool for monitoring and managing Tourette’s disorder.

A limitation of this study is that the study population was from a Grade A tertiary hospital in Jilin Province, and the sample was not representative enough; our findings need to be verified further in various regions of the country. Additionally, most of the participants were from urban areas, and the findings may be influenced as such. Finally, this study was a cross-sectional analysis, so the criterion validity could not be tested based on this sample size. Additionally, we only used Pearson correlation for the association analyses due to the small sample size, which precluded conducting multiple regression analyses. Multiple regression analyses would be necessary to draw firm conclusions about the influence of comorbid ADHD, depression, anxiety, and other symptoms. Therefore, we recommend further testing of the C-IPUTS’ reliability and validity in a larger sample to allow for more comprehensive assessment in future research.

In future studies, we can increase the sample size and age range to investigate the differences in various dimensions of PUs at different age levels. It has been shown that the severity of PUs is associated with reduced connectivity between the cerebral cortex and the primary sensorimotor cortex, while the inferior frontal gyrus is associated with the putamen and insula (48). Therefore, longitudinal studies can be performed using the C-IPUTS, the C-PUTS, and both neurophysiological and neuroimaging tools, which may help obtain some of the neurophysiological markers or endophenotypes of PUs. The age span can also be further expanded to investigate the potential dimensions of the neural phenomena of TDs and PUs.

In summary, the C-PUTS is a reliable and valid brief self-report assessment instrument with good psychometric properties for quantifying the PUs phenomenon. The C-IPUTS is measured by clinicians to assess the number, frequency, intensity, and body parts of PUs in the past week, providing more important supplementary information for the C-PUTS. The C-PUTS and C-IPUTS have good correlations with the symptoms of TD, and there is a slight difference between the two in the comorbidities of TD. This study revealed that the combination of the C-PUTS and C-IPUTS is an effective method for the thorough assessment of PUs, providing a strong basis for the diagnosis and treatment of PUs in TD.

## Data availability statement

The raw data supporting the conclusions of this article will be made available by the authors, without undue reservation.

## Ethics statement

The studies involving humans were approved by the Ethics Committee of the Second Hospital of Jilin University. The studies were conducted in accordance with the local legislation and institutional requirements. Written informed consent for participation in this study was provided by the participants’ legal guardians/next of kin. Written informed consent was obtained from the individual(s), and minor(s)’ legal guardian/next of kin, for the publication of any potentially identifiable images or data included in this article.

## Author contributions

GC and WR carried out the study, analyzed the data, and wrote the manuscript with support from all other authors. ZZ and JT contributed to the implementation of the research and data analysis. YZ and JZ performed the assessment of TD comorbidities. PL designed and directed the research and the manuscript. JM provided the critical feedback and helped shape the manuscript. All authors reviewed and agreed on the final manuscript.

## Footnotes

1
https://www.r-project.org
